# Early Diagnosis of Consumption

**Published:** 1905-04-15

**Authors:** 


### EARLY DIAGNOSIS OF CONSUMPTION.

A well-known physician has observed that the best thing that can happen to a man with diabetes is not to find it out, and the same might be said with some justice of a number of diseases; but tuberculosis of the lungs belongs so emphatically to the opposite type that no apology is needed for repeated insistence on the value of its early identification. Dr. John Hay,[1] reviewing the means at our disposal of detecting the disease at or near its inception, observes that pulmonary tuberculosis seldom arises without symptoms, though these may be trivial enough to be explained away by uninstructed or careless minds. Of these symptoms cough is one of the most constant and most important: a dry, ineffectual, short cough, persistent for any length of time, should always arouse suspicion and suggest an intermittent estimation of the weight. If such a cough is combined with loss of flesh the presumption of tuberculosis is increased, even in the absence of physical signs, and demands investigation of the body-heat, for pyrexia at some time or other during the day, particularly in the afternoon, is an almost constant accompaniment of pulmonary tuberculosis. Hæmoptysis, if the pulmonary derivation of the blood is beyond question, is nearly conclusive of tuberculous disease in the absence of mitral stenosis; and tachycardia with normal temperature is a suggestive combination, and of bad omen if the case should prove to be tuberculous.

The author points out how much the estimate of the probabilities, *pro* and *con*, is assisted by a careful inquiry into collateral incidents, such, for instance, as the family tendencies in this regard, the presence or absence of a possible infecting source in the patient's house or room, the hygienic conditions under which he lives, and the possible association of predisposing diseases, such as influenza.

Passing to the examination of the patient Dr. Hay insists that localisation of signs is of more value than their quality; that negative evidence is to be viewed with profound distrust; that it is unwise to base a diagnosis on any one sign, and that due weight must be given to the normal variations from the normal, so to speak, which are compatible with soundness of the lungs.

Of the actual physical signs a diminished expansion of the apex is among the earliest and most important. It is manifested by an abnormal depth of the supra- and infra-clavicular fossæ, and may be detected manually in a yet earlier stage by standing behind the patient and resting the fingers of each hand on the anterior aspect of the corresponding side of the chest. Vocal resonance and vocal fremitus are habitually greater on the right side than on the left, in health, but a sinistral excess is almost always pathological. As regards auscultation, the first essential is to make the patient breathe properly; to carry out, that is to say, a deep, quiet inspiration by the help of the diaphragm, instead of indulging in the spasmodic jerky contraction of the intercostal muscles which is so favourite a practice with patients. This achieved, adventitious sounds should be sought in the first place below the clavicle in front: when present, they are of the utmost importance, whether pleural or intra-pulmonary, and, if limited to this region, almost diagnostic; as, indeed, is any catarrh constantly localised at the apex. Next to the apex in front, the most likely spot at which to find evidence of tuberculosis is the apex of the lower lobe behind, at a point, that is to say, level with the third dorsal vertebra and its neighbourhood.

Examinations of the sputum, where any can be obtained, are of course urgently called for, nor must absence of tubercle bacilli be accepted as proof positive that the lesion is not tuberculous. The investigation, if negative, must be frequently repeated before any considerable weight can be attached to its findings. Finally, in an obscure case, great help may be derived from a good radiogram of the suspected chest, interpreted by a thoroughly competent observer.

[1] Liverpool Med.-Chir. Jour., Jan. 1905.

